# Women with Symptoms Suggestive of ADHD Are More Likely to Report Symptoms of Iron Deficiency and Heavy Menstrual Bleeding

**DOI:** 10.3390/nu17050785

**Published:** 2025-02-24

**Authors:** Beth MacLean, Paige Buissink, Vernon Louw, Wai Chen, Toby Richards

**Affiliations:** 1 School of Medicine, University of Western Australia, Perth 6009, Australia; 2 Curtin Medical School, Curtin University, Perth 6845, Australia; 3 Division Clinical Haematology, Department of Medicine, University of Cape Town and Groote Schuur Hospital, Cape Town 7701, South Africa; 4 Fiona Stanley Hospital, Perth 6150, Australia; 5 School of Health, Sport and Bioscience, University of East London, London E16 2RD, UK

**Keywords:** iron deficiency, reproductive age women, attention

## Abstract

Background/Objectives: Iron deficiency has been suggested as a potential mechanism for attention-deficit hyperactivity disorder (ADHD) development due to involvement in neurotransmitter synthesis and transporter expression. As iron deficiency is particularly common in women of reproductive age, often due to heavy menstrual bleeding (HMB), we aimed to explore the relationship between iron deficiency, HMB and ADHD in women. Methods: We screened women (18–49 years) at university and local sporting events in Western Australia. To screen for ADHD, section A of the Adult ADHD Self-Report Scale-V1.1 (ASRS-V1.1) and the Adult Concentration Inventory were used to assess cognitive disengagement syndrome (CDS) symptoms. Risk factors for iron deficiency, such as HMB, commonly reported symptoms and a fingerpick haemoglobin concentration (Hb) (Hemocue Hb801) were recorded. Results: Of the 405 completed questionnaires, the mean age was 24.8 ± 10.1 years, the mean Hb was 136.8 ± 12.4 g/L and 6.4% of women were anaemic. Symptoms suggestive of ADHD were reported by 174/405 (43%) women, and 128/405 (32%) women reported HMB. There was a greater prevalence of HMB reported in those experiencing symptoms suggestive of ADHD (39% vs. 26%, *p* = 0.01). Symptoms of fatigue, dizziness, brain fog, anxiety, heart palpitations, headaches, restless legs and depression were more common in patients with symptoms suggestive of ADHD (*p* ≤ 0.01) and HMB (*p* < 0.05). Anaemia status did not influence ADHD status (*p* = 0.87) nor CDS scores (15.7 ± 7.0 vs. 13.8 ± 6.1, *p* = 0.17). Conclusions: There is an apparent relationship between those with symptoms reported in ADHD, HMB and iron deficiency. Further exploration is required to determine whether there is a causative relationship.

## 1. Introduction

Attention-deficit hyperactivity disorder (ADHD) is a neurodevelopmental disorder characterised by symptoms of inattention and/or hyperactivity and impulsivity, which present during childhood and persist into adulthood [1,2]. Behavioural symptoms include forgetfulness, fidgeting and disorganisation [2]. According to the Diagnostic and Statistical Manual of Mental Disorders (5th ed., text rev.; DSM-5-TR), there are three clinical presentations of ADHD [3]: predominantly inattentive symptoms, predominantly hyperactive–impulsive symptoms and a combined presentation (i.e., both inattentive and hyperactive–impulsive symptoms). Individuals with ADHD are more likely to experience occupational, academic and interpersonal difficulties [4]. Furthermore, individuals with ADHD are at a significantly greater risk of multiple medical conditions and reduced life expectancy [5].

ADHD is estimated to occur in approximately 2.5–6% of the adult population [1,5,6,7]. During childhood, the ratio of boys to girls diagnosed with ADHD is approximately 3:1; however, in adulthood, the ratio becomes approximately 2:1 to 1:1 [8]. Within adult community samples, the prevalence of ADHD is similarly lower in females (3.2%) in comparison to males (5.4%) (note, in this paper, the terms “female” and “women” will be used interchangeably to discuss those of the female sex) [9]. Sex differences in the presentation of ADHD symptoms are proposed to contribute to the discrepancy in prevalence. Males are more likely to demonstrate predominantly hyperactive-impulsive or combined symptom presentations, whereas females are more likely to demonstrate predominantly inattentive symptoms [9,10]. Inattentive symptoms, in comparison to hyperactive-impulsive symptoms, are less noticeable and therefore less likely to be diagnosed [8]. Therefore, differences in prevalence may reflect underdiagnosis in females, and ADHD may affect men and women at least equally [10].

The DSM-5-TR categorically defines ADHD by the number of symptoms present across several different settings, which have been present before 12 years of age and significantly interfere with daily functioning. ADHD is diagnosed in clinical practice using a categorical definition; however, symptoms or traits of ADHD can be commonly observed in the general population. Acros-Burgos and Acosta reported that approximately 60% of the general population may demonstrate any symptom of inattention or hyperactivity [11]. Therefore, ADHD can be conceptualised as the extreme end of a continuum of traits present in the general population [12]. Vogel et al. reported that although a greater number of symptoms or ADHD-related traits in the general population were associated with difficulties in daily functioning, even low numbers of symptoms were associated with an increased disease burden [12]. Therefore, although a low number of symptoms may not necessitate a diagnosis of ADHD, this does not discount the need for clinical attention that these symptoms require.

### Iron Deficiency as a Proposed Mechanism for ADHD Development

Currently, there is no single known cause of ADHD; however, a combination of risk factors has been proposed, including prenatal and perinatal factors, socioeconomic factors and genetic predispositions [2,5,13,14,15,16]. A possible causal mechanism of ADHD is suggested to involve deficits in the regulation of the neurotransmitter dopamine. This is because dopamine is involved in psychomotor activity and executive functions, which are characteristically impaired in ADHD [15].

Iron deficiency has also been proposed as a potential risk factor for the development of ADHD. In iron-deficient cohorts of infants, children and women, there appears to be a greater susceptibility to developing symptoms of ADHD [15,17,18,19], while conversely, a greater incidence of iron deficiency has been found in cohorts of children with ADHD [20]. A potential link has been suggested between low maternal ferritin, ferritin being the protein that stores bodily iron during gestation and an increased likelihood of the offspring developing ADHD symptoms during infancy and childhood [13].

Mechanistically, iron is essential for many neurocognitive processes. This can be observed in iron-deficient cohorts, where neurocognitive symptoms are frequently reported, including depression, anxiety, difficulty concentrating and the patient-coined term ‘brain fog’ [21,22]. Neurocognitively, iron is involved in nerve myelination, oxygen transport to the brain, neurotransmitter synthesis and transporter expression, with the latter two incorporating dopamine synthesis and dopamine transporter (DAT) expression and, as such, is currently proposed as the most plausible mechanism for iron deficiency to trigger ADHD symptoms [23].

Women are at an increased risk of developing an iron deficiency during their reproductive years [24]. Heavy menstrual bleeding (HMB) is the most common aetiology of iron deficiency in women during their reproductive years, as heavier periods can result in almost double the amount of iron lost per period in comparison to those with regular menstrual flow [24]. The taboo nature of discussions regarding menstrual flow implements a rate-limiting factor to HMB diagnosis; however, HMB is estimated to affect between 25 and 53% of menstruating women [24]. Despite the hypothesis regarding the influence of iron deficiency on symptoms of ADHD, no studies have explored a potential link between HMB, as a common risk factor for iron deficiency development, and symptoms of ADHD. As a systemically underappreciated condition in women [8], we wished to further explore the presentation of ADHD symptoms in women and to explore whether HMB and iron deficiency could play a role in symptom development.

## 2. Materials and Methods

### 2.1. Assessing Symptoms of ADHD and CDS

We screened otherwise healthy women at university and sporting events across Perth, Western Australia. We included non-pregnant women between the ages of 18 to 49 years. The primary outcome of the study was to identify the prevalence of ADHD symptoms in reproductive-aged women. Section A of the WHO Adult ADHD Self-Report Scale-V1.1 (ASRS-V1.1 Screener) questionnaire was used to identify participants with symptoms highly consistent with ADHD in adults, and for the purpose of the study, those meeting the corresponding threshold criteria as defined by the World Health Organisation (WHO) will be classified as the ADHD group, though we acknowledge that this tool does not necessitate diagnosis [25]. Details for the classification of the ADHD group are outlined in Table 1. The 6-item screener is a shortened version of the ASRS-V1.1, comprising items derived from the 18-item DSM-5-TR (Diagnostic and Statistical Manual of Mental Disorders, 5th ed., text rev) checklist that best psychometrically predicts ADHD [26]. The screener was developed based on stepwise logistic regression and is suggested to outperform the extended ASRS-V1.1, with validation studies in primary care and university settings reporting sensitivity to 66–100%, specificity to 0.71–99.5%, a positive predictive value (PPV) of 89.3%, internal consistency of 0.54–0.72 and test re-test reliability from 0.58 to 0.77 with the latter maintained when controlling for gender, ethnicity, age and psychiatric co-morbidities [25,26,27,28,29]. Responses were coded using the corresponding guidance into categorical variables of those with ADHD symptoms and those without. It should be noted that when applied to psychiatric care, the 6-item screener was not as successful in distinguishing ADHD from non-ADHD psychiatric patients, producing a greater incidence of false positives [30].

The Adult Concentration Inventory (ACI) self-report questionnaire was used to assess symptoms of cognitive disengagement syndrome (CDS) symptoms, note the latter has been previously known as sluggish cognitive tempo (SCT) [31]. The ACI is a 16-item self-report rating scale that is designed to screen for symptoms of CDS. It was developed following a meta-analysis which identified CDS symptoms that are consistently distinct from the symptoms of ADHD [32]. The results from the meta-analysis suggested 13 specific CDS items that capture both cognitive and behavioural symptoms of CDS with the addition of 3 further items to measure mental confusion [32]. Responses were reported as a continuous score.

### 2.2. Assessing Iron Deficiency Risk and Anaemia

To facilitate mass measurement, haemoglobin concentration (Hb) was assessed in a point-of-care method using fingerprick testing (Hemocue Hb801), with anaemia status then determined according to WHO guidelines for identifying iron deficiency in adult women (Hb < 120 g/L) [33]. An iron deficiency risk factor questionnaire was created using commonly reported symptoms of iron deficiency identified from previous studies [34], and a 4-item screener for heavy menstrual bleeding (HMB), which was developed by Fraser et al., was used to detect symptoms of HMB [22,35] (full questionnaire available in Supplementary Table S1).

Participants with two or more of the following symptoms during a typical menstrual cycle were classified as having HMB:

- Flooding through clothes or bedding;
- Need for frequent changes of sanitary towels or tampons (meaning changes every 2 h or less, or 12 sanitary items per period);
- Need of double sanitary protection (tampons and towels);
- Pass large blood clots.

### 2.3. Sample Size

The sample size was calculated using a confidence level of 95% with a Z score of 1.96 and a margin of error of 5%. The population size of women between the ages of 18 to 49 in Western Australia was estimated to be 1.076 million, according to the Australian Bureau of Statistics 2022 [36]. With any ADHD symptom estimated to be reported in 60% of the population [11], we calculated a minimum sample size of 369 women.

### 2.4. Statistical Analysis

Complete case analysis was used. Data were analysed as those with/without HMB and ADHD symptoms and by anaemia status. Continuous variables were reported as mean ± standard deviation and analysed using independent samples *t*-test. Pearson’s chi-square was used to compare the prevalence of iron deficiency symptoms by those with/without ADHD symptoms and HMB status. In addition, linear regression analysis was used to assess the relationship between haemoglobin concentration and CDS score.

### 2.5. Ethical Considerations

Informed consent was obtained from participants, with ethical approval granted by the Human Ethics Office at the University of Western Australia.

## 3. Results

**Figure 1:**
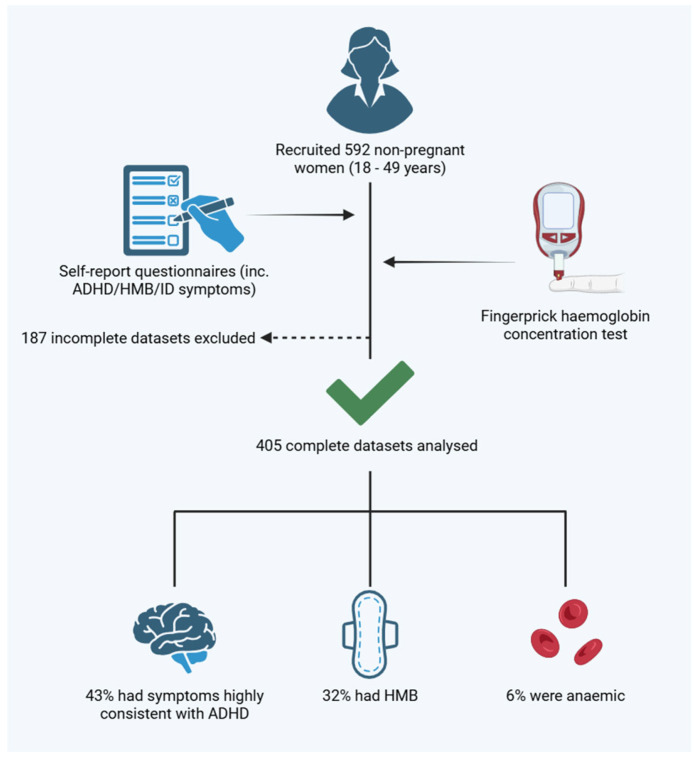
Study summary.

Between February to May 2022, we recruited 592 participants, of which, 187 questionnaires were incomplete, leaving 405 datasets for analysis (illustrated in Figure 1). The mean age of the cohort was 24.8 ± 10.1 years. Overall, 174/405 (43.0%) of women reported symptoms highly consistent with ADHD in accordance with the thresholds defined by the ASRS-V1; for the purpose of analysis, this group will further be referred to as the ADHD symptoms group.

The average Hb in the cohort was 136.8 ± 12.4 g/L, with 26/405 (6.4%) having an Hb consistent with anaemia (Table 2). Heavy menstrual bleeding (HMB) symptoms were reported by 128/405 (31.6%) women. Those who reported HMB did not show a difference in Hb (136.5 ± 12.6 vs. 137.0 ± 12.3, 0.742). Those women with HMB reported more symptoms of iron deficiency (Table 3). Similarly, the prevalence of iron deficiency symptoms reported was analysed by anaemia status; however, anaemia status did not influence the prevalence of iron deficiency symptoms (*p* > 0.05; Supplementary Table S2).

**Figure 2:**
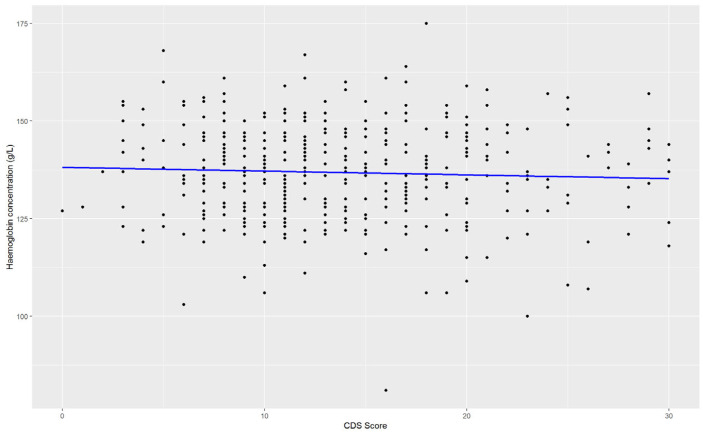
Haemoglobin concentration against CDS score. Linear regression analysis showed multiple R-squared: 0.0022, adjusted r-squared: −0.001989, F test = 0.9197 and *p* = 0.3381.

Overall, the ADHD symptoms group were younger on average (22.7 ± 7.8 years vs. 26.3 ± 11.3 years, *p* < 0.01). They reported a higher incidence of HMB (39.1% vs. 26.0%, *p* = 0.01) and reported a higher mean CDS score (17.6 ± 6.0 vs. 11.1 ± 4.6, *p* < 0.01). Symptoms of iron deficiency were also more commonly reported in those with ADHD symptoms, including brain fog, anxiety and depression (*p* ≤ 0.01, Table 2). The group with ADHD symptoms did not show a difference in Hb (136.9 ± 12.5 g/L vs. 136.7 ± 12.3 g/L, *p* = 0.87). Linear regression analysis found no relationship between Hb and CDS score (*p* = 0.338, Figure 2)

## 4. Discussion

From the results of the study, we can observe that symptoms of ADHD appear to be common in women during their reproductive years. Notably, the younger cohort was more likely to report symptoms of ADHD and HMB. Those reporting ADHD symptoms or HMB were more likely to experience symptoms associated with iron deficiency. Though these data are limited in terms of identifying the specificity of the relationship, it leads us to question whether the relationship is causative or whether there is simply a large overlap in the presentation of iron deficiency and ADHD. The cross-sectional nature of the study becomes a limitation, and therefore, a longitudinal study regarding these outcomes may provide better insight into a causative relationship.

Naturally, the study was limited by the lack of point-of-care testing available for ferritin as a marker of iron deficiency. Anaemia status is useful as, when making the assumption that over half of anaemia cases globally are the result of an iron deficiency [37], it allows us to observe the influence of iron deficiency at the extreme end of manifestation. However, as likely reflected in the lack of relationship between anaemia with both iron deficiency symptoms and HMB status in the current study, anaemia screening may not be a useful indicator of iron deficiency in this cohort. Furthermore, this method excludes the detection of non-anaemic iron deficiency; therefore, the next steps to exploring a relationship between iron deficiency and ADHD would require ferritin assessment, as the latter is required to determine iron deficiency diagnosis [24]. Notably, fingerprick haemoglobin concentration testing is beneficial for mass testing, though future work may consider the use of venous tests to ensure greater accuracy in haemoglobin concentration measurement and, consequently, anaemia detection [38].

In terms of ADHD assessment, the distinct association of higher CDS scores in the ADHD symptoms group suggest both questionnaires are in agreement regarding ADHD identification. Notably, the prevalence of those with symptoms highly consistent with ADHD was substantial (43%), reflecting previous work that suggests 60% of the population display any symptom of ADHD [11]. Though notably, these symptoms do not always necessitate a diagnosis, with only around 3% of women expected to have an ADHD diagnosis [6,9]. To address this and the risk of bias associated with self-report questionnaires, future work may consider the use of the gold standard interview process to diagnose ADHD [25,39].

In terms of iron deficiency risk factors, the study does not account for birth control, menstrual frequency, cycle timing, menopause, reproductive conditions or tranexamic acid use, which are all factors which can influence iron status and menstrual flow [24]. Furthermore, the population included in the study were reproductive-aged women at university and sporting events, which may lead to over-representation of younger, healthier and more physically active participants, the latter of which has been associated with attenuation of ADHD symptoms [40,41]. Future work should aim to recruit community-based women of reproductive age to reduce biases. In addition, future reporting should provide further insight into co-morbidities, another psychological diagnosis, socioeconomic background and race, which are factors that have been associated with ADHD diagnosis [4,5,30,42,43,44].

Genetic studies have supported that ADHD symptoms present as a continuum within the general population [16]; even when ADHD symptoms are not severe enough to meet the criteria for diagnosis, the symptoms in the general population still warrant investigation. Though ADHD is less commonly diagnosed in women, these data highlight that menstrual health may not be appropriately questioned, recognised, or acted on in the diagnostic algorithm. It may be of value to implement screening for HMB and iron deficiency prior to ADHD investigation in women during their reproductive years, as both are easy to diagnose and treat and should not be overlooked as potential causative factors of neurological symptoms in women during their reproductive years.

## 5. Conclusions

There is an apparent relationship between those with symptoms suggestive of ADHD, HMB and symptoms commonly reported in iron deficiency. Further exploration of the association between ferritin, HMB and ADHD symptoms is required to explore the nature of the relationship.

## Figures and Tables

**Table 1:** ASRS-v1.1 screener. The above table demonstrates the signal questions for determining those with symptoms suggestive of ADHD. Those with four or more responses in the shaded area of the chart were deemed to have symptoms of ADHD in accordance with the official tool instructions [25].

Question	Never	Rarely	Sometimes	Often	Very Often
How often do you have trouble wrapping up the final details of a project once the challenging parts have been done?					
How often do you have difficulty getting things in order when you have to do a task that requires organisation?					
How often do you have problems remembering appointments or obligations?					
When you have a task that requires a lot of thought, how often do you avoid or delay getting started?					
How often do you fidget or squirm with your hands or feet when you have to sit down for a long time?					
How often do you feel overly active and compelled to do things, like you were driven by a motor?					

**Table 2:** Demographics and symptoms of iron deficiency by ADHD status. The above table compares those with symptoms suggestive of ADHD, according to ASRS-V1.1 Screener, with those who did not (non-ADHD). Continuous data are reported as mean ± standard deviation, and prevalence data are reported as the number of responders with the percentage of the group they comprise.

Symptoms	ADHD	Non-ADHD	Total	*p* Value
n	174	231	405	-
Mean Age (years)	22.7 (±7.8)	26.3 (±11.3)	24.8 (±10.1)	**<0.01**
Hb (g/L)	136.9 (±12.5)	136.7 (±12.3)	136.8 (±12.4)	0.87
HMB	68 (39.1%)	60 (26.0%)	128 (31.6%)	**0.01**
Anaemia	12 (6.9%)	14 (6.1%)	26 (6.4%) 26 (6.4%)	0.89
Mean CDS Score	17.6 (±6.0)	11.1 (±4.6)	13.9 (±6.1) 13.9 (±6.1)	**<0.01**
Fatigue/Exhaustion	155 (89.1%)	181 (78.4%)	336 (83.0%)	**0.01**
Dizziness/Fainting	130 (74.7%)	128 (55.4%)	258 (63.7%)	**<0.01**
Brain Fog	121 (69.5%)	96 (41.6%)	217 (53.6%)	**<0.01**
Anxiety	123 (70.7%)	92 (39.8%)	215 (53.1%)	**<0.01**
Feeling Cold	59 (33.9%)	60 (26.0%)	119 (29.4%)	0.10
Shortness of Breath	76 (43.7%)	59 (25.5%)	135 (33.3%)	**<0.01**
Heart Palpitations	57 (32.8%)	38 (16.5%)	95 (23.5%)	**<0.01**
Headaches	105 (60.3%)	104 (45.0%)	209 (51.6%)	**<0.01**
Hair Loss	50 (28.7%)	47 (20.3%)	97 (24.0%)	0.07
Restless Legs	69 (39.7%)	58 (25.1%)	127 (31.4%)	**<0.01**
Depression	69 (39.7%)	27 (11.7%)	96 (23.7%)	**<0.01**

Significant *p* values (*p* < 0.05) are indicated in bold.

**Table 3:** Demographics and symptoms of iron deficiency by HMB status. The above table compares those with symptoms of HMB, according to the 4-item screener by Fraser et al. [35], with those who did not (non-HMB). Continuous data are reported as mean ± standard deviation, and prevalence data are reported as the number of responders with the percentage of the group they comprise.

Symptoms	HMB	Non-HMB	*p* Value
n	128 (31.6%)	277 (68.4%)	-
Mean Age (years)	22.9 (±7.2)	25.6 (±11.1)	**0.003**
Hb (g/L)	136.5 (±12.6)	137.0 (±12.3)	0.742
Anaemia	7 (2.5%)	19 (6.9%)	0.754
Mean CDS Score	15.5 (±5.7)	13.2 (±6.2)	**<0.001**
ADHD	68 (53.1%)	106 (38.3%)	**0.007**
Fatigue/Exhaustion	118 (92.2%)	218 (78.7%)	**0.001**
Dizziness/Fainting	92 (71.9%)	166 (59.9%)	**0.027**
Brain Fog	82 (64.1%)	135 (48.7%)	**0.006**
Anxiety	87 (68.0%)	128 (46.2%)	**<0.001**
Feeling Cold	44 (34.4%)	75 (27.1%)	**0.017**
Shortness of Breath	51 (39.8%)	84 (30.3%)	0.076
Heart Palpitations	40 (31.3%)	55 (19.9%)	**0.017**
Headaches	76 (59.4%)	133 (48.0%)	**0.043**
Hair Loss	32 (25.0%)	65 (23.5%)	0.833
Restless Legs	51 (39.8%)	76 (27.4%)	**0.017**
Depression	45 (35.2%)	51 (18.4%)	**<0.001**

Significant *p* values (*p* < 0.05) are indicated in bold.

## Data Availability

Upon reasonable written request, data sharing will be considered.

## Supplementary Materials

The following supporting information can be downloaded at https://www.mdpi.com/article/10.3390/nu17050785/s1. Table S1. Questionnaire; Table S2. Analysis by anaemia status.

## Abbreviations

  The following abbreviations are used in this manuscript:

ADHD	Attention-deficit hyperactivity disorder
HMB	Heavy menstrual bleeding
ASRS-V1.1	Adult ADHD Self-Report Scale-V1.1
CDS	Cognitive disengagement syndrome
Hb	Haemoglobin concentration
DSM-5-TR	Diagnostic and Statistical Manual of Mental Disorders 5th ed., text rev
DAT	Dopamine transporter
WHO	World Health Organisation
PPV	Positive predictive value
ACI	Adult Concentration Inventory
SCT	Sluggish cognitive tempo
