# Supplementary material for: Women with Symptoms Suggestive of ADHD Are More Likely to Report Symptoms of Iron Deficiency and Heavy Menstrual Bleeding
Source: Nutrients. 2025 Feb 24;17(5):785. doi: 10.3390/nu17050785 (PMC11902013; doi:10.3390/nu17050785)
Supplement: Supplementary file 1 [file nutrients-17-00785-s001.zip › nutrients-3462838-supplementary.pdf]

Supplementary Materials

Table S1

Questionnaire

Start of Block: Personal Details

*

Q1 Please provide some personal details.

What is your age?

____

Q2 Which sex describes you best?

- Female (1)
- Male (2)
- Prefer not to say (3)

End of Block: Personal Details

Start of Block: Mensuration

Display This Question:

If Which sex describes you best? = Female

Q3 In regards to your period, have you ever experienced any of the following? (Please select all that apply)

- ☐ Flooding through clothes or bedding (1)
- ☐ Need of frequent changes of sanitary towels or tampons (meaning changes every 2 hours or less, or 12 sanitary items per period). (2)
- ☐ Need of double sanitary protection (tampons and towels) (3)
- ☐ Pass large blood clots (4)
- ☐ None of the above (5)

End of Block: Mensuration

Start of Block: Iron Deficiency Symptoms and Impact

Q4 Below are a list of symptoms- What are your symptoms of iron deficiency? (Please pick all that apply to you)

- ☐ Fatigue/Exhaustion (1)
- ☐ Dizziness/Feeling Light-Headed/Fainting (2)
- ☐ Brain Fog (inability to think clearly, forgetful, foggy) (3)
- ☐ Anxiety (4)
- ☐ Feeling Cold (5)
- ☐ Shortness of breath (air hunger, out of breath, puffed easily) (6)
- ☐ Heart palpitations (thumping heart, rapid pulse, high pulse) (7)
- ☐ Headaches (9)
- ☐ Hair loss (10)
- ☐ Restless legs while at rest or while sleeping (14)
- ☐ Depression (17)

End of Block: Iron Deficiency Symptoms and Impact

Start of Block: SCT

Q5 Please use the scale below to indicate how strongly the following statements apply to you

	Not at all (1)	Sometimes (2)	Often (3)	Very often (4)
I stare off into space (1)	○	○	○	○
I feel sleepy or drowsy during the day (2)	○	○	○	○
I lose my train of thought (3)	○	○	○	○
I get lost in my own thoughts (4)	○	○	○	○
I get tired easily (5)	○	○	○	○
I feel confused (6)	○	○	○	○
I zone out or space out (7)	○	○	○	○
I daydream (8)	○	○	○	○
I forget what I was going to say (9)	○	○	○	○
My mind gets mixed up (10)	○	○	○	○

Q6 Similarly, use the new scale below to indicate how strongly the following statements apply to you

	Never (1)	Rarely (2)	Sometimes (3)	Often (4)	Very Often (5)
How often do you have trouble wrapping up the final details of a project, once the challenging parts have been done? (1)	○	○	○	○	○
How often do you have difficulty getting things in order when you have to do a task that requires organization? (2)	○	○	○	○	○
How often do you have problems remembering appointments or obligations? (3)	○	○	○	○	○
When you have a task that requires a lot of thought, how often do you avoid or delay getting started? (4)	○	○	○	○	○
How often do you fidget or squirm with your hands or feet when you have a sit down for a long time? (5)	○	○	○	○	○
How often do you feel overly active and compelled to do things, like you were driven by a motor? (6)	○	○	○	○	○

End of Block: SCT

Start of Block: Block 6

*

Q7 Once you receive your haemoglobin concentration from the finger prick test, please input your result below (just the numbers no unit)

____

End of Block: Block 6

Table S2
Analysis by anaemia status

Symptoms	Anaemic	Non-Anaemic	Total	*p value*
n	26	379	405	-
Mean Age (years)	24.5 (± 8.7)	24.8 (± 10.2)	24.8 (± 10.1)	0.89
Hb (g/L)	110.9 (± 8.5)	138.6 (± 10.5)	136.8 (± 12.4)	**<0.01**
HMB	7 (26.9%)	121 (31.9%)	128 (31.6%)	0.75
ADHD	12 (46.2%)	162 (42.7%)	174 (43.0%)	0.89
Mean CDS Score	15.7 (± 7.0)	13.8 (± 6.1)	13.9 (± 6.1)	0.17
Fatigue/Exhaustion	21 (80.8%)	315 (83.1%)	336 (83.0%)	0.97
Dizziness/Fainting	15 (57.8%)	243 (64.1%)	258 (63.7%)	0.65
Brain Fog	13 (50.0%)	204 (53.8%)	217 (53.6%)	0.86
Anxiety	18 (70.7%)	197 (52.0%)	215 (53.1%)	0.13
Feeling Cold	10 (69.2%)	109 (28.8%)	119 (29.4%)	0.41
Shortness of Breath	9 (34.6%)	126 (33.2%)	135 (33.3%)	1.00
Heart Palpitations	5 (19.2%)	90 (23.7%)	95 (23.5%)	0.77
Headaches	14 (53.8%)	195 (51.5%)	209 (51.6%)	0.97
Hair Loss	5 (19.2%)	92 (24.3%)	97 (24.0%)	0.73
Restless Legs	6 (23.1%)	121 (31.9%)	127 (31.4%)	0.47
Depression	3 (11.5%)	93 (24.5%)	96 (23.7%)	0.20
